# Preliminary Evaluation of an Automated Blood Cell Analyzer for Its Use with Blood Samples from Rainbow Trout *Oncorhynchus mykiss*

**DOI:** 10.3390/ani15091265

**Published:** 2025-04-29

**Authors:** Montse Mesalles, Meritxell Uroz, Irene Brandts, Emmanuel Serrano, Rafaela Cuenca, Josep Pastor, Mariana Teles

**Affiliations:** 1 Servei d’Hematologia Clínica Veterinària (SHCV), Departament de Medicina i Cirurgia Animals, Facultat de Veterinària, Universitat Autònoma de Barcelona (UAB), 08193 Cerdanyola del Vallès, Barcelona, Spain; montse.mesalles@uab.cat (M.M.); rafaela.cuenca@uab.cat (R.C.); 2 Departament de Biologia Celular, Fisiologia i Immunologia, Facultat de Biociencies, Universitat Autònoma de Barcelona (UAB), 08193 Cerdanyola del Vallès, Barcelona, Spain; meritxell.um@gmail.com (M.U.); irene.brandts@uab.cat (I.B.); 3 Wildlife Ecology & Health Group (WE&H), Servei d’Ecopatologia de Fauna Salvatge (SEFaS), Departament de Medicina i Cirurgia Animals, Facultat de Veterinària, Universitat Autònoma de Barcelona (UAB), 08193 Cerdanyola del Vallès, Barcelona, Spain; emmanuel.serrano@uab.cat

**Keywords:** fish health, hematology, hematological methods validation, blood automated analyzer, rainbow trout

## Abstract

Hematological studies provide essential information about the health of animals, which is crucial for veterinary medicine, scientific research, and aquaculture. Automatic hematological analyzers are an alternative to manual methods, offering faster and more reliable results. The objective of this study was to validate the Sysmex XN-1000V automatic hematology analyzer for blood samples from rainbow trout (*Oncorhynchus mykiss*), examine the effects of two anticoagulants (K_2_EDTA and lithium heparin), and establish normal blood reference values for this fish species. Additionally, comparative studies were conducted between the Sysmex XN-1000V and manual methods (hemocytometer cell count and blood smear estimation), and reference intervals were established. Ninety-nine heparinized blood samples were analyzed for validation and sample stability tests. The results showed extremely good precision, with a coefficient of variation (CV) below 3% for RBCs, HGB, and HCT and less than 5% for non-RBC cells (leukocytes plus thrombocytes). However, heterophils (%) exhibited higher variability, with a CV of 15.08%. Linearity was excellent, and the carry-over was below 1% for all parameters. The sample stability test indicated that samples could be analyzed for up to 48 h when stored at 4 °C and up to 24 h at room temperature. Non-RBC cells were the first to degrade over time. The automated and manual methods demonstrated good correlation and agreement, validating the analyzer’s accuracy. The effects of two anticoagulants, K_2_EDTA and lithium heparin, on the blood samples were also studied. Heparin was the preferred anticoagulant for routine hematological analysis of rainbow trout blood with the Sysmex XN-1000V analyzer. In conclusion, the Sysmex XN-1000V enables complete hemogram analyses to be performed quickly and accurately, standardizing techniques, harmonizing results, and providing reliable reference intervals with *O mykiss* blood.

## Simple Summary

This study aimed to validate the Sysmex XN-1000V hematology analyzer for rainbow trout (*Oncorhynchus mykiss*) blood samples, compare it with manual methods, and establish normal blood reference values. The blood cell analyzer showed high precision and low variability for most parameters, except heterophils. The analyzer demonstrated excellent linearity and minimal carry-over. Sample stability tests indicated that blood samples could be stored for up to 48 h at 4 °C and 24 h at room temperature, with non-RBCs degrading. Heparin was found to be the preferred anticoagulant to be used with the analyzer. This study concluded that the Sysmex XN-1000V provides quick, accurate, and reliable hematological analyses, standardizing techniques and harmonizing results for rainbow trout blood, which is valuable for veterinary medicine, scientific research, and aquaculture.

## 1. Introduction

Blood cell parameters have been widely used as a diagnostic tool to assess the health status and welfare of fish in response to changes related to nutrition, water quality, and pathological conditions [1,2,3,4,5,6].

Hematological analysis of fish blood is usually performed by manual methods, which have been criticized for their imprecision, inaccuracy, and turnaround time [7]. Despite their limitations, they remain the option of choice in fish and other species with nucleated erythrocytes and thrombocytes, where automated analysis determination is not optimized, making it challenging to replace manual methods. The growth of aquaculture, as well as increasing concerns about the pollution of aquatic ecosystems, have highlighted the need to develop accurate automated methods to improve the quality and speed of obtaining hemogram results in fish [8].

Rainbow trout (*Oncorhynchus mykiss*) is one of the most significant species in global aquaculture. Known for its rapid growth, high survival rate, and remarkable adaptability to diverse aquatic conditions, it has become an invaluable resource for the industry. This species is cultivated extensively worldwide, playing a crucial role in meeting the growing demand for sustainable and high-quality protein sources [9]. Additionally, its sensitivity to various pollutants in aquatic environments has garnered significant interest from the scientific community, making it a valuable biomarker for assessing ecosystem health [10].

Blood analysis in aquaculture is limited due to the time and specialized expertise required for manual methods. Additionally, data interpretation is challenging due to the absence of standardized protocols and the physiological variability among species. Hematologic automated analyzers present significant advantages compared with manual techniques, offering time- and cost-effective options, eliminating operator-tied errors, and consequently increasing the repeatability and reliability of the obtained results [11,12,13,14,15,16,17]. Despite the potential for preventing economic losses in the aquaculture sector and potentially revolutionizing preventive fish medicine, their validation and use in fish hematology remain limited, probably due to a lack of validation with manual methods.

External and internal factors contribute significantly to fish interspecies variability, ecological diversity, and specific physiological characteristics [11,12,18,19,20,21,22,23,24]. These factors in turn affect the hematological parameters in the species, making it difficult to establish normal hematological reference intervals in fish, which are essential for the diagnosis of various pathophysiological conditions [25,26] and have been established for most mammalian species. Furthermore, the absence of standardized methodologies for cell counting in fish species contributes to the complexity of interpreting published data for diagnostic applications [27,28,29,30,31]. Therefore, the use of automated analytical techniques to evaluate fish blood can help improve our understanding of fish hematology and, consequently, the effectiveness of fish farm management.

This study aimed to (1) evaluate the performance (including assessment of the precision, linearity, carry-over, and stability) of the PLT-F channel and the bird settings of the Sysmex XN-1000V hematology analyzer (Sysmex Corporation, Kobe, Japan) on rainbow trout blood samples and comparing the obtained results with those obtained by manual methods; (2) evaluate the effects of two commonly used anticoagulants (ethylenediaminetetraacetic acid dipotassium salt (K_2_EDTA) and lithium heparin (Li-heparin)) on trout blood parameters; and (3) establish reference intervals for rainbow trout using ASVCP guidelines [32,33].

## 2. Materials and Methods

### 2.1. Animals

This study used 99 adult rainbow trout (weight: 63 g ± 12 g; length: 17 ± 2 cm) from a local fish farm at Molinou Rialb (northeast Spain). The animals were considered healthy based on an external examination to detect some abnormality or infestation. The fish were transported to the facilities of the Universitat Autònoma de Barcelona (AQUAB-Fish) and acclimated before the experiment for 4 weeks in a closed recirculating freshwater system with a 1000 L tank at a density of 8.4 kg/m^3^. A constant water temperature (15 °C) and quality were maintained, with continuous monitoring of the dissolved oxygen (<9 mg/L), pH (within the range of 6.0–8.5), nitrite (<0.05 mg/L), nitrate (<1.0 mg/L), and total ammonia (<0.07 mg/L) levels. Throughout the study, fish were subjected to a photoperiod of 12L:12D and were fed a daily maintenance diet equaling 3% of their body weight (“Trouw Nutrition” S.A.U., Madrid, Spain). During the experimental period, water was exchanged to maintain optimal water quality conditions and promote the good condition of the fish in the study.

Before blood sampling, the randomly selected fish were anesthetized with buffered tricaine methane sulfonate (MS-222, Sigma-Aldrich^®^, Merck KGaA, Darmstadt, Germany). at a sublethal dose of 55 mg/L [34]. Blood collection was carried out via caudal venipuncture (always in the morning between 9:00 a.m. and 10:00 a.m.) using 21G needles and 2 mL heparinized insulin syringes and kept in 1.5 mL heparin-coated microtubes. To study the effects of the two different anticoagulants on the blood parameters, 19 samples of the total number were duplicated in two tubes with lithium heparin and K_2_EDTA.

Before analysis, blood samples were placed on an agitator (VSR23, Grant Instruments Ltd., Cambridge, UK) for 10 min. All samples were analyzed once within 2 h of sampling with a Sysmex XN-1000V automated blood cell analyzer (Sysmex Co., Kobe, Japan) and during the morning of the same day of collection with manual methods by the same operator, except for those samples for the storage and stability tests.

All experimental procedures involving fish were submitted and authorized by the Ethics and Animal Care Committee of the Universitat Autònoma de Barcelona (permit numbers OH4218 and DAMM 11251), following the International Guiding Principles for Biomedical Research Involving Animals (EU2010/63).

### 2.2. Instruments and Reference Methods

#### 2.2.1. Automated Hematology Analyzer

The automated hematology analyzer evaluated in this study (Sysmex XN-1000V) is an advanced automated hematology system already used and validated for mammals based on fluorescence flow cytometry and an impedance analysis system (multispecies software 3.05). The analyzer’s novel PLT-F channel incorporates dedicated optical-fluorescent platelet analysis using Fluorocell PLT fluorescent dye (Sysmex Co., Kobe, Japan) that provides not only the total platelet count but also useful information on the immature platelet fraction. The PLT-F channel combined with the bird profile of the analyzer allows determination of the different nucleated blood cell populations (RBC, WBC, and PLT) of non-mammalian animal species.

The default bird profile provided by the analyzer can be adapted to other species with similar hematological characteristics, such as fish, which facilitates the development of new profiles. In our study, the PLT-F channel combined with the bird profile of the analyzer was used to create a trout profile. To increase the reliability of the results and minimize errors caused by the subjective differentiation of cell populations in fish, particularly due to the similarity between the predominant lymphoid cells and thrombocytes, a manual adjustment of the scatter plot cells from the automated analyzer was performed. Cells were classified into two groups: erythroid cells (RBCs) and non-erythroid cells (non-RBCs). Within the non-RBC region of the PLT-F scatter plot, and using the extended manual gate function, we could distinguish two distinct populations for further analysis: the cluster of mononuclear cells, representing lymphocytes, monocytes, and thrombocytes, and the heterophil cluster with greater complexity and granularity.

**Figure 1:**
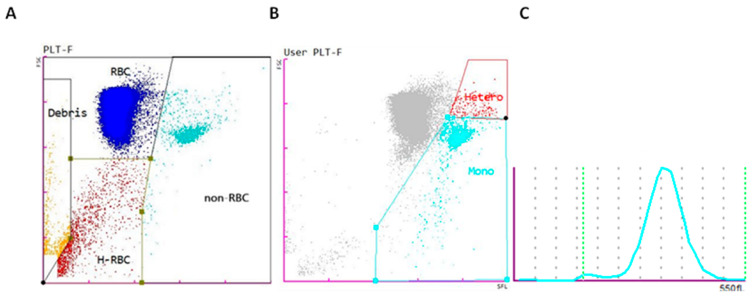
(**A**) Scatter plot of the PLT-F channel of the Sysmex XN-1000V’s rainbow trout profile of the different blood cell populations: red blood cells (RBCs) (blue), non-erythrocyte cells (non-RBCs) (turquoise blue), hemolyzed red blood cells (H-RBCs) (red), and debris (orange). (**B**) Scatter plot showing the manual gating adjustment of the mononuclear (Mono) and heterophil (Hetero) cells. (**C**) Histogram of the mean corpuscular volume of erythrocytes.

Figure 1 illustrates these two primary populations (RBCs and non-RBCs), emphasizing the separation between mononuclear cells and heterophils, which enables a more precise and detailed analysis of each cell type.

The analyzer also automatically calculates parameters such as the hematocrit (Hct), mean corpuscular volume (MCV), mean corpuscular hemoglobin (MCH), mean corpuscular hemoglobin concentration (MCHC), and red cell distribution width, expressed as both a coefficient of variation (RDW-CV) and standard deviation (RDW-SD).

Daily internal quality control (QC) was conducted using Level 2 or a normal range of commercially available QC material (Sysmex XN Check Level 2; Sysmex Corporation).

#### 2.2.2. Manual Count of Rainbow Trout Blood Samples

For the RBC and non-RBC counts, the manual chamber counting method was chosen as the “reference method” in this study. A 1:200 dilution of blood was prepared (5 µL heparinized blood mixed with 995 µL commercial Natt and Herrick diluent (Bioanalytic GmbH, Umkirch, Germany). The mixture was incubated at room temperature (25 °C) for 3–5 min on an automated mixer to achieve optimal staining. Cells were allowed to settle in the chambers for an additional 1–2 min. RBC and non-RBC counting were performed in duplicate at a 40× magnification. RBCs in 5 squares consisting of 80 small squares (0.2 mm^2^) were enumerated in each chamber, and the average of the two counts was calculated and multiplied by 10,000 to determine the total RBC count per millimeter cubed. The non-RBCs in 9 squares consisting of 144 small squares (9 mm^2^) were enumerated in each chamber, and the average of the two counts was calculated and multiplied by 200 to obtain the total non-RBC count per millimeter cubed.

The packed cell volume (PCV) was determined through the microhematocrit method. Two capillary tubes were centrifuged at 13,143 g for 5 min (Centromix II BL, J.P Selecta S.A., Barcelona, Spain), and the mean of the two measurements was calculated. The plasma protein concentration was also determined using the same capillary tubes and a clinical refractometer (Euromex, Arnhem, The Netherlands).

Two blood smears were prepared for each sample and stained with a modified Romanowsky stain (Panoptic, QCA S.A., Tarragona, Spain). The estimated non-RBC count per millimeter cubed was obtained by calculating the mean number of non-RBCs per high dry field (40× magnification) in 10 consecutive fields. The mean was multiplied by 1600 to obtain the final non-RBC count. A differential non-RBC count was performed by examining 100 non-RBCs at a 100× magnification with an optical microscope (Eclipse 50i, Nikon Corporation, Tokyo, Japan). Cells were classified as mononuclear or heterophils.

### 2.3. Sysmex XN-1000V Validation for Rainbow Trout Blood Samples

To validate the Sysmex XN-1000V for its use with rainbow trout blood, the following parameters were measured: RBC count, hemoglobin concentration, Hct, MCV, non-RBC count, percentage, and number of heterophils and mononuclear cells.

#### 2.3.1. Precision

The precision of the instrument was determined by analyzing 2 selected heparinized rainbow trout blood samples (one with high values and one with normal values) in triplicate. The precision of the manual method was assessed for RBC and non-RBC counts by two observers, one more experienced than the other, who received previous training. The procedure was performed in triplicate by analyzing one blood sample from dilution to counting.

#### 2.3.2. Linearity

Duplicate measurements of a 6-point serial dilution (100%, 75%, 50%, 25%, 12.5%, 6.25%) with Sysmex diluent, Cellpack-DCL (Sysmex Co., Kobe, Japan) from a concentrated heparinized blood sample (centrifuging the blood at 1600× *g* for 10 min and removing 80% of the plasma) were evaluated. The instrument’s diluent was analyzed in triplicate to demonstrate that it did not produce interferences or variations in the amount of each parameter analyzed.

#### 2.3.3. Carry-Over

Carry-over was assessed by measuring one concentrated blood sample (high level) in triplicate followed by a low-level sample (obtained using Sysmex diluent solution, Cellpack-DCL, (Sysmex Co., Kobe, Japan).

#### 2.3.4. Stability Test

Stability was determined by using four animals. Heparinized blood samples were divided into two aliquots and stored at 4 °C and room temperature (25 °C). Samples were then analyzed in duplicate by the same operator immediately and at 4, 6, 24, 48, and 72 h. Before each analysis, blood samples stored at 4 °C were placed on a blood mixer for 10 min for rewarming.

### 2.4. Agreement Between Manual and Automated Methods

Ninety-nine adult rainbow trout blood samples were used in the comparative study between automated and manual methods. The following parameters were compared: RBC count (automated vs. manual); Hct/PCV; non-RBC count (automated vs. manual; automated vs. smear estimation; and manual vs. smear estimation); and heterophil and mononuclear percentages (automated vs. smear estimation).

### 2.5. Reference Intervals

Reference intervals (RIs) were established using 63 out of the 99 blood samples collected. The handling and evaluation of samples were conducted following the methodology previously described for this study.

### 2.6. Statistical Analysis

Blood parameter normality was assessed using the Shapiro–Wilk test (*p* < 0.05), applying parametric tests to parameters with a normal distribution and non-parametric tests to parameters without a normal distribution. The coefficients of variation (CVs) as percentages, mean, and standard deviation were calculated for precision for each parameter. Linearity was assessed by linear regression analysis (Pearson’s correlation (r)). The effect of storage was assessed using Friedman’s test with Dunnett’s multiple comparisons. Spearman’s linear correlation, Passing–Bablok non-parametric regression, and Bland–Altman analysis were used to compare the Sysmex XN-1000V and manual results. An RI was defined under the ASVCP guidelines [33], the population was defined using the mean, median, minimum, and maximum, as well as a reference interval using the 5th and 95th percentiles, since some parameters showed a Gaussian distribution and others did not. The influence of anticoagulants (Li-heparin or K_2_EDTA) on hematological parameters was assessed by the paired Wilcoxon non-parametric test. The statistical program GraphPad Prism v 8.0.1 (GraphPad Software, Sant Diego, CA, USA) was used for statistical comparisons, where *p* < 0.05 was considered statistically significant, while Passing–Bablok regression and Bland–Altman analysis were performed with https://bahar.shinyapps.io/method_compare/ accessed on 8 November 2024. Statistical significance was considered at α = 0.05.

## 3. Results

### 3.1. Precision

The precision obtained with the hematology analyzer for low-to-normal and high cell counts was good for all parameters except for the heterophil count (Table 1). The precision of the manual method for RBCs and non-RBCs, performed by two observers, showed high variability, especially for the RBC count, with a coefficient of variation >20% (Table 2).

### 3.2. Linearity

**Figure 2:**
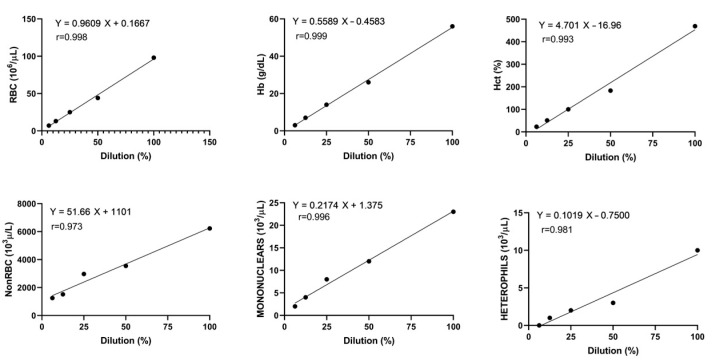
Linearity of red blood cells (RBCs), hemoglobin concentration (Hb), hematocrit (Hct), non-erythrocyte cells (non-RBCs), mononuclear cells, and heterophils of one rainbow trout blood sample with Pearson’s correlation and regression line.

The linearity of the Sysmex XN-1000V was excellent for the RBC count, Hb, Hct, and mononuclear count, with correlation coefficients higher than 0.99, and acceptable for the non-RBC and heterophil counts, with correlation coefficients of 0.97 and 0.98, respectively (Figure 2).

### 3.3. Carry-Over

All of the results for carry-over were in the range provided by the manufacturer (<1%).

### 3.4. Stability

**Figure 3:**
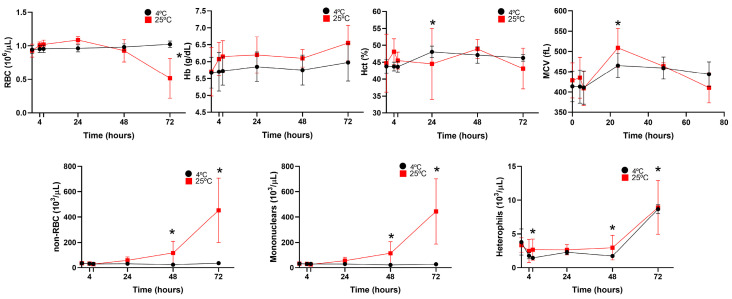
Stability of four *O. mykiss* blood samples after storage at 4 °C (black circles) and 25 °C (red squares), determined by the Sysmex XN-1000V analyzer. Red blood cell (RBC); hemoglobin concentration (Hb); hematocrit (Hct); mean corpuscular volume (MCV); non-erythrocyte cell (non-RBC); and mononuclear and heterophil count data are presented as mean and standard deviation. Statistical significance is indicated by an asterisk (*p* < 0.05).

**Figure 4:**
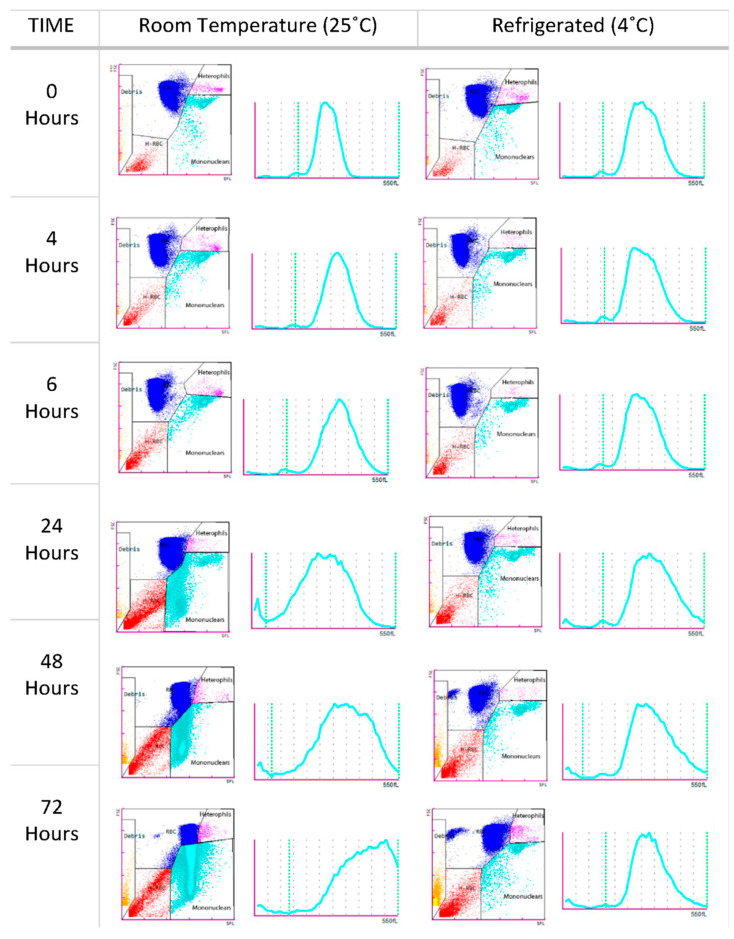
Scatter plots and histograms of an *O. mykiss* blood sample from the stability study analyzed by the Sysmex XN-1000V at different times and temperatures (25 °C and 4 °C), showing RBCs (blue), heterophils (pink), mononuclear cells (turquoise blue), hemolyzed erythrocytes (red), and debris (orange).

The effects of blood storage in heparinized samples at 25 °C and 4 °C are presented in Figure 3 and Figure 4. RBC counts remained stable for up to 48 h at room temperature, decreasing steadily thereafter. At 4 °C, the counts remained stable for up to 72 h. Storage did not significantly affect the Hb or Hct values, which remained stable at both temperatures. MCV values increased over time, with significant differences observed at 24 h in the samples stored at 25 °C. After this point, the values began to decrease, likely due to hemolysis. This trend was not observed in the samples stored at 4 °C. Non-RBC counts increased significantly 48 h after blood collection in the samples stored at room temperature, possibly due to erythrocyte hemolysis (Figure 4). However, samples stored at 4 °C maintained a stable non-erythrocyte cell count. Mononuclear and heterophil counts remained stable at both 25 °C and 4 °C for up to 48 h. After this point, a significant increase in both cell types was observed in the samples stored at both temperatures.

### 3.5. Agreement Between Automated Blood Cell Analyzer and Manual Methods

**Figure 5:**
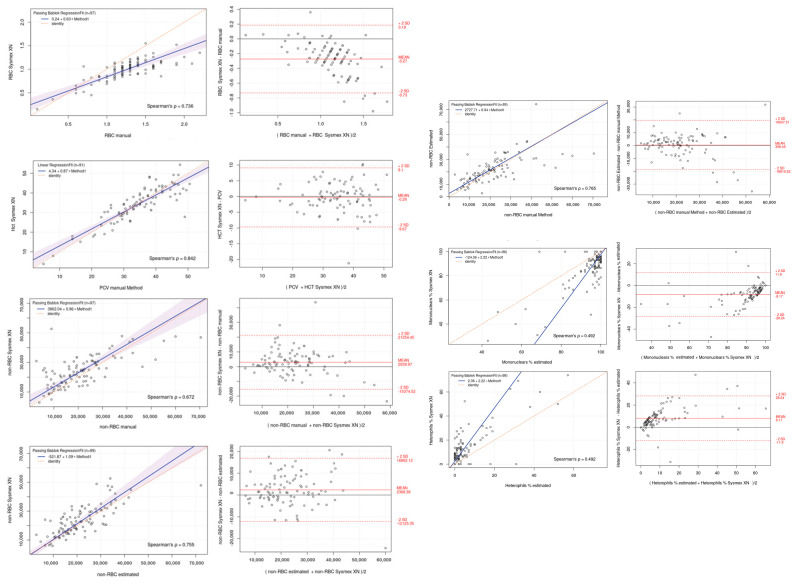
Passing–Bablok regression (**left**) and Bland–Altman plots (**right**) of Sysmex XN-1000V automated counting versus manual methods for red blood cell (RBC), hematocrit/PCV (Hct/PCV), non-erythrocyte cell (non-RBCs), mononuclear, and heterophil percentages. Passing–Bablok plots show the dispersion of the points around the regression line and the 95% confidence interval of the regression line, except for the mononuclear and heterophil counts due to the high dispersion present. Bland–Altman plots show the mean difference (bias, solid line) between the two methods and the 95% upper and lower limits of agreement (dashed line).

The Passing–Bablok regression analysis showed good correlation and agreement between the results obtained with the Sysmex XN-1000V, the cell counts found with the manual method from a hemocytometer, and the cell estimation of the blood smear for all parameters, as shown in the graphs (Table 3 and Figure 5). The Bland–Altman plot for RBCs and hematocrit values showed a proportional and negative bias between the two measurement methods. The non-RBC population showed a proportional and positive bias, regardless of the measurement method, whether determined by an automated system or estimated from the blood smear. In contrast, the heterophils showed a random and positive bias, while the mononuclear percentage showed a random and negative bias.

### 3.6. Effect of the Anticoagulant

**Figure 6:**
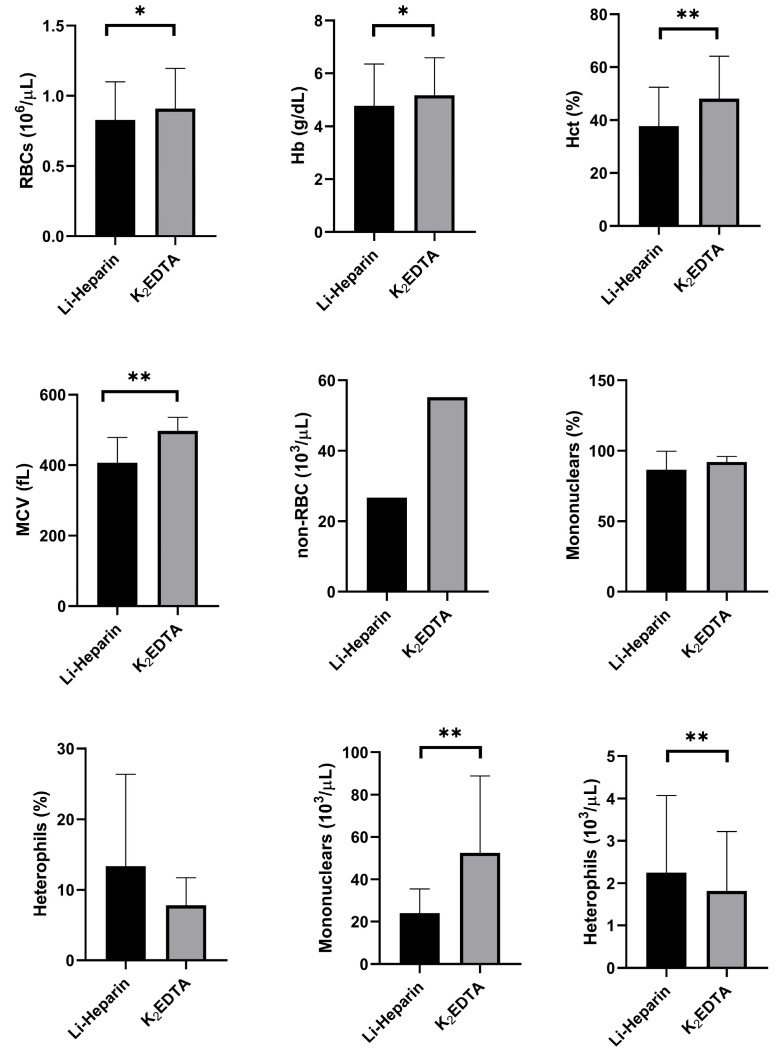
Effects of two types of anticoagulants (Li-heparin and K_2_EDTA) on hematological parameters of 19 rainbow trout, expressed as mean and standard deviation, showing red blood cells (RBCs), hemoglobin concentration (Hb), hematocrit (Hct), mean corpuscular volume (MCV), and non-erythrocyte cells (non-RBCs). Statistical significance is indicated by an asterisk (* *p* < 0.05 and ** *p* < 0.01).

Figure 6 illustrates the effects of two different anticoagulants (Li-heparin and K_2_EDTA) on the hematological parameters of rainbow trout, as analyzed by the automated hematology analyzer. In all cases (*n* = 19), except for heterophils, the blood samples preserved with K_2_EDTA showed significantly higher RBC counts, Hb concentrations, Hct percentages, MCV values, non-RBC counts, and mononuclear counts compared with those preserved with Li-heparin. The type of anticoagulant did not significantly affect the percentages of mononuclear cells or heterophils.

**Figure 7:**
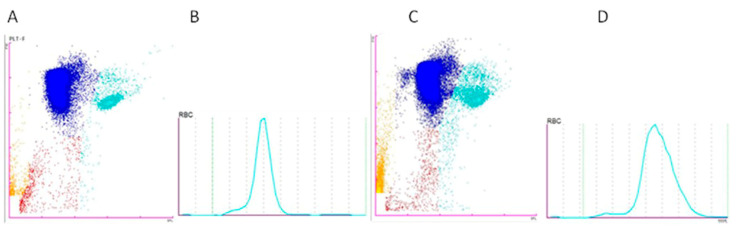
Scatter plots and histograms of a blood sample collected with Li-heparin ((**A**) PLT-F scatter plot and (**B**) MCV distribution histogram) and K_2_EDTA ((**C**) PLT-F scatter plot and (**D**) MCV distribution histogram), showing red blood cells (RBCs) (blue), non-erythrocyte cells (non-RBCs) (turquoise blue), hemolyzed red blood cells (H-RBCs) (red), and debris (orange).

The Sysmex XN-1000V scatter plot analysis of the samples showed differences depending on the anticoagulant used. The samples preserved with Li-heparin exhibited greater RBC scatter plot compaction and better differentiation between the different cell populations. Conversely, the samples preserved with K_2_EDTA showed more cell debris, likely due to artifact-induced cell lysis, making it more difficult to differentiate the population of non-RBCs. Moreover, the histogram provided by the analyzer for the erythrocytes reflected an increase in the MCV due to red cell swelling induced by K_2_EDTA (Figure 7).

### 3.7. Reference Intervals (RIs)

Table 4 summarizes the reference intervals for hematology analytes obtained with the Sysmex XN-1000V analyzer compared to the results obtained through manual methods.

## 4. Discussion

The evaluation of automated blood cell analyzers for fish blood samples has remained a relatively underdeveloped area due to their distinctive hematological characteristics, which pose a considerable challenge in adapting these machines originally designed for mammalian blood samples. Scientific publications on the use of automated methods for species with nucleated erythrocytes and thrombocytes are scarce [25,35], and the scientific community still relies on manual methods for analyzing the blood of these species, despite their inherent limitations. The emergence of advanced analyzers, such as the Sysmex XN-1000V, offers promising prospects for automating blood cell counting in non-mammalian species (birds, reptiles, and amphibians), which was highlighted in a recent study by Meazzi et al. [36] focusing on *Testudo hermanni* samples. To the authors’ knowledge, this is the first study that evaluates the use of the Sysmex XN-1000V in heparinized whole blood of rainbow trout.

The first part of this study focused on evaluating the performance of the PLT-F channel and the bird settings of the Sysmex XN-1000V (Sysmex Corporation, Kobe, Japan) hematology analyzer on *O. mykiss* teleost fish blood samples and comparing the obtained results with those obtained by manual methods. According to the validation process results, based on ASVCP guidelines [32] and the International Committee for Standardization in Hematology’s [37] recommendations, the Sysmex XN-1000V hematology analyzer showed excellent linearity and good precision for most of the parameters tested (CVs < 5%) but not for the heterophil count (CV > 15%). This may have occurred for two reasons. First, this type of cell is present in an extremely low percentage of teleost fish, which makes small variations in the count have a greater impact [38]. Second, the fact that the heterophil count is not a predefined parameter in the Sysmex XN-1000V and it is required to manually adjust the events cloud in the analyzer can introduce inaccuracies, depending on the operator’s expertise and training, especially when dealing with low cell numbers.

Sample stability is defined as the capability of a sample to retain the initial value of a measured quantity for a defined period and storage conditions [39]. It may vary based mainly on the analyzer used (different analyzers use different techniques to characterize and quantify hematological analytes) but also the species, the analyte tested, the anticoagulant used, or the storage conditions [40,41]. The results of this study show that refrigerated samples exhibit greater stability compared with those stored at room temperature, as the cold environment preserves cellular structures and delays degradation processes [42]. In the present study, we observed a statistically significant increase in MCV at 24 h after collecting samples stored at room temperature, which was mostly due to decreased membrane resistance and a subsequent increase in cell permeability during in vitro storage, which induced erythrocyte swelling in a time-dependent manner [43,44,45,46] and subsequent lysis [47].

Hemoglobin and hematocrit values remained consistent throughout the 72 h storage period, contrasting with findings reported by Fazio et al. [46]. These differences are likely attributable to variations in experimental methodologies and reagents. In this study, the non-RBC counts in the blood samples stored at room temperature remained stable for up to 48 h post-collection. Beyond this period, a significant increase in the non-RBC counts was observed, likely due to hemolysis of erythroid cells and degradation of non-erythroid cells. Such degradation is exacerbated by prolonged storage at room temperature and at 4 °C beyond 48 h, resulting in cellular degeneration that compromises the analyzer’s ability to accurately distinguish between erythroid and non-erythroid cells (mononuclear cells, heterophils, and thrombocytes). Furthermore, the presence of cellular debris plus the remaining nuclei after lysis of RBCs may have artificially increased the non-erythrocyte cell count in our study.

Controlled studies have shown that refrigerated avian blood deteriorates within 12 h regardless of the anticoagulant [48], whereas reptilian blood specimens are stable for 24 h [49]. Consistent with the recommendations of these authors, we advocate for the prompt processing of blood samples for hematological analysis in rainbow trout. Specifically, when non-RBC counts are assessed, samples stored at room temperature should be analyzed within 24 h of collection to ensure accuracy. Even when refrigerated, analysis should be performed within 48 h to minimize the risk of result misinterpretation due to sample degradation.

Comparisons between the analyzer and manual methods revealed a slight underestimation of RBC and Hct values, alongside a marginal overestimation of non-RBC counts. These discrepancies likely stem from the inherent inaccuracies of the manual method, coupled with the subjective interpretation required for the analyzer’s scatter plots. Differentiating between thrombocytes and lymphocytes is particularly challenging [38], as immature thrombocytes (round) may be misclassified as lymphocytes, thus falling into the mononuclear population category [7]. Similar to other researchers [50,51], we assert that classifying thrombocytes within the mononuclear population yields more reliable results, reducing confounding variables and enhancing consistency. Although the methods are not interchangeable, their overall agreement supports their continued use for comparable clinicopathological interpretations in future studies.

Since fish blood clots quickly, particularly in stressed individuals, anticoagulants are crucial for sample collection. Various studies have demonstrated that the hematological characteristics of fish species can differ depending on the type of anticoagulant used [7]. That is why we chose to test the effects of two types of anticoagulants (Li-heparin and K_2_EDTA) on the hematological parameters of rainbow trout obtained by the automated analyzer. In our study, Li-heparin has proved to be the most suitable anticoagulant for preserving rainbow trout blood, and only a slight decrease in the RBC count and hemoglobin concentration was observed, likely due to the dilution effect of liquid heparin. Heparin does not cause hemolysis, which led to it being considered a safer anticoagulant by some authors, although in some fish species, it does not appear to be effective in preventing blood clotting [52,53,54].

Although EDTA is preferable for certain fish species due to its ability to maintain hematological values more consistently over time [19,55], it appears to have a more pronounced impact on hematological parameters, particularly in red blood cells, in most fish species compared with other anticoagulants. Thus, Hattingh [56] observed that EDTA caused hemolysis in fish species and always increased the cell volume. Tavares-Dias and Sandrim [35] reported that EDTA reduced Hct and Hb values when compared with heparin. According to Witeska and Wargocka, [57], EDTA-induced erythrocyte swelling causes cell membrane disruption and hemolysis followed by gradual karyolysis. In addition, some studies have shown that EDTA causes osmotic fragility and, consequently, hemolysis of these cells, increases the hemoglobin content, and decreases the red blood cell count in fish blood samples treated with this anticoagulant.

In our study, cell populations were not properly differentiated in the scatter plots in the K_2_EDTA-treated samples due to cell lysis and debris, resulting in artefactually increased non-erythrocyte cell counts. Moreover, K_2_EDTA induced RBC swelling, which in turn increased the MCV and hematocrit values. Our findings are consistent with those previously reported [1,17,18,47,57,58] and highlight the importance of selecting an appropriate anticoagulant for hematological studies in fish that minimizes alterations and provides more accurate hematological results [51].

Accurate interpretation of hemograms obtained using the Sysmex XN-1000V requires specific hematologic reference values [36]. In this study, we established, for the first time, reference intervals (RIs) for rainbow trout blood samples analyzed with the XN-1000V alongside manual reference methods (hemocytometer and smear estimation). Our results were consistent across methods and aligned with previously published values obtained through manual and automated impedance-based systems [29,31,46,59,60]. Despite this, variations were observed, reflecting the impact of methodological differences as well as preanalytical and analytical conditions, as noted by Ahmed et al. [61]. These findings emphasize the importance of establishing laboratory-specific reference intervals to ensure accurate and reliable hematological evaluations.

## 5. Conclusions

In this work, the XN-1000V automated analyzer was validated for heparinized rainbow trout blood samples, which represents a significant advancement in fish hematology research and diagnostics. This system reduces the inherent variability associated with manual methods and provides more reliable and accurate results.

## Figures and Tables

**Table 1:** Precision results study for one *Oncorhynchus mykiss* blood sample with low-to-normal and one with high cell counts determined by the Sysmex XN-1000V.

	Low–Normal		High	
Parameter (Unit)	Mean	CV (%)	Mean	CV (%)
**RBC (×10^6^/µL)**	0.64	2.71	1.21	0.83
**Hb (g/dL)**	4.03	1.43	5.87	3.94
**Hct (%)**	26.87	1.07	58.57	1.78
**MCV (fL)**	361.5	1.39	ND	ND
**Non-RBC (×10^3^/µL)**	29.34	4.51	52.06	4.22
**Mononuclear (%)**	87.07	2.24	ND	ND
**Heterophils (%)**	12.93	15.08	ND	ND

Coefficient of variation (CV); red blood cells (RBCs); hemoglobin concentration (Hb); hematocrit (Hct); mean corpuscular volume (MCV); non-erythrocytes cells (non-RBCs); not determined (ND).

**Table 2:** Total red blood cell and non-RBC counts were determined manually by two observers for one *O. mykiss* blood sample.

	Observer 1		Observer 2		Observer 1 + 2
Parameter (Unit)	Median	CV (%)	Median	CV (%)	CV (%)
**RBCs (×10^6^/µL)**	1.28	5.63	1.77	15.68	21.27
**Non-RBCs (×10^3^/µL)**	19.20	12.01	17.10	23.94	17.57

Red blood cells (RBCs); non-erythrocyte cells (non-RBCs); coefficient of variation (CV).

**Table 3:** Results of the comparison between the XN-1000V analyzer and manual methods of rainbow trout blood samples.

		Correlation		Limit of Agreement 95% (Bias)			
Parameter (Method)	N	Rs Spearman	Slope (IC 95%)	Intercept (IC 95%)	Mean	Low Limit	Upper Limit
RBCs (×10^6^/µL) (Sysmex XN-Manual)	97	0.74	0.6 (0.5–0.71)	0.24 (0.11–0.37)	−0.27	−0.73	0.19
Hct/PCV (%) (Sysmex XN-PCV)	91	0.84	1.01 (0.9–1.12)	−0.24 (−3.5–4.09)	−0.29	−9.67	9.1
Non-RBCs (×10^3^/µL) (Sysmex XN-Manual)	97	0.67	0.96 (0.76–1.11)	3862 (892.8–8262)	2939.97	−15,374.5	21,254.4
Non-RBCs (×10^3^/µL) (Sysmex XN-Smear estimation)	99	0.76	1.09 (0.93–1.27)	−521.87 (−3817.81–2958)	2368.38	−12,125.3	16,862.1
Non-RBCs (×10^3^/µL) (Manual-Smear estimation)	95	0.765	0.94 (0.31–0.81)	2727.71 (−284.11–6055.16)	398.49	−18,810.5	19,607.5
Mononuclear (%) (SysmexXN-Smear estimation)	98	0.49	2.22 (1.77–3.34)	−124.36 (−234.5–80.61)	−8.17	−28.2	11.9
Heterophils (%) (Sysmex XN-Smear estimation)	98	0.49	2.22 (1.7–3.34)	2.36 (0–3.38)	8.17	−11.9	28.2

Red blood cells (RBCs), hematocrit (Hct), packed cell volume (PCV), non-erythrocytes (non-RBCs), and mononuclear cells (lymphocytes, monocytes, and thrombocytes).

**Table 4:** Reference intervals for adult rainbow trout CBC determined by the Sysmex XN-1000V analyzer (n = 63) and manual counts (hemocytometer and blood smear estimation).

Parameter	Unit	Dist. (Method)	Mean ± SD	Median Range (Min–Max)	Reference Interval (95%CI)	Out of RI (n/lot (%))
	Automated Method (Sysmex XN-1000V)					
**RBC**	×10^6^/µL	G (SUD)	1.03 ± 0.18	1.04 (0.65–1.55)	0.761–1.33	3/63 (4.8)
**Hb**	g/dL	G (SUD)	6.07 ± 1.01	6 (3.9–8.8)	4.33–7.69	2/63 (3.2)
**Hct**	%	G (SUD)	46.3 ± 8.92	46.3 (23.6–68.6)	31.84–60.27	2/63 (3.2)
**MCV**	fL	G (SUD)	412.57 ± 49.74	410.1 (308–540)	331.02–488.02	3/63 (4.8)
**MCH**	pg	G (SUD)	54.39 ± 6.05	53.8 (38.9–73.8)	45.68–66.71	5/63 (7.9)
**MCHC**	g/dL	G (SUD)	13.34 ± 1.93	13.2 (8.8–18.2)	11.2–16.58	5/63 (7.9)
**RDW-SD**	fL	G (SUD)	38.79 ± 12.36	37.5 (18.4–73.2)	20.35–58.52	1/63 (1.6)
**Non-RBC**	×10^3^/µL	G (SUD)	25.98 ± 11.54	25.31 (17.08–47.74)	13.35–44.63	3/63 (4.8)
**RDW-CV**	%	G (SUD)	13.04 ± 3.29	13 (7.1–20.2)	7.99–18.3	2/63 (3.2)
**Heterophils**	%	NG (RUD)	7.05 ± 10.84	3 (0–52)	4.31–9.78	3/63 (4.8)
**Heterophils**	×10^3^/µL	NG (RUD)	10.80 ± 7.22	9.86 (0–25)	4.9–23.82	13/63 (20.6)
**Mononuclear**	%	NG (RUD)	84.62 ± 15	88.9 (30.8–100)	79.05–100	3/63 (4.8)
**Mononuclear**	×10^3^/µL	NG (RUD)	22.53 ± 11.79	21.86 (3.30–53.32)	13.35–44.63	13/63 (20.6)
	Manual Method (Hemocytometer)					
**RBC**	×10^6^/µL	G (SUD)	1.34 ± 0.34	1.32 (0.57–2.22)	0.791–1.94	3/63 (4.8)
**PCV**	%	G (SUD)	36.68 ± 7.41	36.5 (21–50.5)	24.1–48.4	1/63 (1.6)
**Non-RBC**	×10^3^/µL	NG (RUD)	22.99 ± 14.22	21.80 (2.8–70.40)	5.84–51.26	8/63 (12.7)
**TP**	g/dL	G (SUD)	3.96 ± 0.86	4 (1.7–5.3)	2.65–5	2/63 (3.2)
	Manual Method (Smear Estimation)					
**Non-RBC**	×10^3^/µL	NG (RUD)	21.65 ± 11.54	19.04 (2.88–72.16)	13.6–37.69	19/63 (30.1)
**Mononuclear**	%	NG (RUD)	92.95 ± 10.84	97 (48–100)	68.1–100	4/63 (6.3)
**Mononuclear**	×10^3^/µL	NG (RUD)	20.17 ± 11.19	18 (2.4–68.55)	11.91–36.60	19/63 (30.1)
**Heterophils**	%	NG (RUD)	7.05 ± 10.84	3 (0–52)	1–31.90	11/63 (17.4)
**Heterophils**	×10^3^/µL	NG (RUD)	1.49 ± 2.55	0.56 (0–12.52)	0.21–8.28	19/63 (30.1)

RBC = red blood cell count; Hb = hemoglobin concentration; Hct = hematocrit; MCV = mean corpuscular volume; MCH = mean corpuscular hemoglobin; MCHC = mean corpuscular hemoglobin concentration; non-RBC = non-erythrocytes cell count; RDW-SD and RDW-CV = standard deviation and coefficient of variation of erythrocyte distribution width, respectively; PCV = packed cell volume. Values are shown as mean ± SD; median range (minimum-maximum); and reference interval 95% confidence interval. Method: SUD = standard untransformed data; RUD = robust untransformed data; Out of RI = out of reference intervals; G = Gaussian; NG = Non-Gaussian.

## Data Availability

The data that support the findings of this study are available from the corresponding author upon reasonable request.
